# Pupil and Glint Detection Using Wearable Camera Sensor and Near-Infrared LED Array

**DOI:** 10.3390/s151229792

**Published:** 2015-12-02

**Authors:** Jianzhong Wang, Guangyue Zhang, Jiadong Shi

**Affiliations:** School of Mechatronical Engineering, Beijing Institute of Technology, 5 South Zhongguancun Street, Haidian District, Beijing 100081, China; cwjzwang@bit.edu.cn (J.Z.W.); zhangguangyue@yeah.net (G.Y.Z.)

**Keywords:** wearable camera sensor, pupil detection, glint detection, circular ring rays location, total least squares fitting, Gaussian fitting

## Abstract

This paper proposes a novel pupil and glint detection method for gaze tracking system using a wearable camera sensor and near-infrared LED array. A novel circular ring rays location (CRRL) method is proposed for pupil boundary points detection. Firstly, improved Otsu optimal threshold binarization, opening-and-closing operation and projection of 3D gray-level histogram are utilized to estimate rough pupil center and radius. Secondly, a circular ring area including pupil edge inside is determined according to rough pupil center and radius. Thirdly, a series of rays are shot from inner to outer ring to collect pupil boundary points. Interference points are eliminated by calculating gradient amplitude. At last, an improved total least squares is proposed to fit collected pupil boundary points. In addition, the improved total least squares developed is utilized for the solution of Gaussian function deformation to calculate glint center. The experimental results show that the proposed method is more robust and accurate than conventional detection methods. When interference factors such as glints and natural light reflection are located on pupil contour, pupil boundary points and center can be detected accurately. The proposed method contributes to enhance stability, accuracy and real-time quality of gaze tracking system.

## 1. Introduction

Human beings acquire 80%~90% of outside information from our eyes. Humans’ visual perception information can be acquired through eye gaze tracking. With the increasing development of computer/machine vision technology, gaze tracking technology has been more and more widely applied in fields of medicine [1], production tests [2], human-machine interaction [3,4], aviation military [5,6], *etc.*

As one of traditional gaze tracking methods [7,8,9,10,11,12], the pupil center-corneal reflection (PCCR) technique has been developed and improved increasingly in recent years [13,14,15,16,17,18]. Pupil and glint (corneal reflection) center detection plays a crucial role on gaze tracking methods based on PCCR. There are always interference factors such as eyelashes, eyelids, shadows and natural light reflection in the images acquired by a CCD camera, which will cause false boundary points around pupil contour. In order to ensure the accuracy of gaze estimation, robust and accurate method of pupil and glint detection is essential.

Previous scholars have done a great many research works on pupil and glint detection. Ebisawa [19] proposes a pupil detection technique using two alternate infrared light sources and image difference of bright and dark eye image. Bright/dark eye image is acquired by switching on light source in coaxial/uncoaxial with the camera during add/even field alternatively, due to which the sampling time is limited. The glint position stays almost fixed. To detect it, pupil brightness should be as low as possible. Although the image difference method is simple, switching on/off of light sources may influence its stability. To overcome the limitation of this technique, methods utilizing single eye image are proposed continuously.

In [13], in order to obtain accurate pupil center position, double ellipse fitting (rough and detailed) are performed to eliminate false boundary points. It is difficult to eliminate false boundary points around pupil contour and double ellipse fitting cost a long time. The glint is detected by searching near the pupil. Its centroid is then calculated as center position. The uncertain searching time and result of glint can lead to instability of the method. Yoo *et al.* [20] acquire rough pupil bound by iterative projection. Snakes are utilized to converge to the boundary of pupil. Elimination of false boundary points is not considered. Glint searching region is limited by rough pupil bound. At last, pupil and glint center position are determined by ellipse fitting. Gwon *et al.* [21] locate approximate pupil area using CED method, then precise pupil center is obtained by calculating geometric center of black pixels. Before pupil detection, glints are erased by neighboring pixels in horizontal direction. The erasion causes error to pixel points around pupil contour and influences accuracy of pupil center location. To better locate pupil boundary, Li *et al.* [22] develop a feature-based method. In the process of feature detection, pupil contour candidates are detected along a series of rays shooting from a best guess of pupil center and marked with crosses. RANSAC is applied to differentiate pupil contour points (inliers) and interference points (outliers). When interference factors such as glints and natural light reflection locate on or around pupil contour, part of interference points and pupil contour points are mixed together. In this case, RANSAC is not capable enough to differentiate them. The location accuracy of pupil center is affected. Krishnamoorthi and Annapoorani [23] propose a boundary extraction technique to localize pupil. Orthogonal polynomials model is adopted to analysis the structure of an eye image. Hartley’s statistical hypothesis test is employed in edge map extraction. A where-to-go approach is proposed to find pupil boundary points with the assistant of weightage assignment. Although the algorithm can locate pupil boundary points accurately, it has a limitation of boundary assumption.

The remainder of this paper is organized as follows: Section 2 presents the proposed method in detail. Section 3 describes the experiments and shows the experimental results. Section 4 concludes the whole work.

## 2. Proposed Method

A novel and robust method of pupil and glint detection using wearable camera sensor and near-infrared LED array for gaze tracking system is proposed in this paper. Compared with original Starburst, the proposed circular ring rays location(CRRL) method has higher stability, accuracy and real-time quality. This method overcomes the location uncertainty of initial shooting point of rays. The process of shooting rays back towards the start point to collect more pupil boundary points is omitted. RANSAC is also omitted for the reason that the interference points can be eliminated effectively. Pupil center can be detected accurately when interference points are located on or around pupil contour. Improved Otsu method is employed to acquire the eye’s binary image. Part of the remainder interference factors (including eyelashes and eyelids) are eliminated by opening-and-closing operation with structure elements of different size. Projections of 3D gray-level histogram are utilized to estimate rough pupil radius and center position. The circular ring area is determined by provisional pupil radius and center. A series of rays with equal gap are shot from the inner to outer ring to detect pupil boundary points by calculating gradient amplitude. Gradient amplitude of each pixel is used to eliminate false boundary points. Spline interpolation is performed on the neighborhood of boundary points to obtain subpixel-precise ones. Improved total least squares is developed to fit ellipse and then pupil center position is calculated through elliptic equation fitted. Because the gray levels of glint pixels are higher than anywhere else, rough glint region is estimated by binarization with a fixed threshold level. According to glint’s illumination intensity (suited for Gaussian distribution), Gaussian function deformation solved by improved total least squares is utilized to calculate glint center.

### 2.1. Proposed Gaze Tracking Device

**Figure 1:**
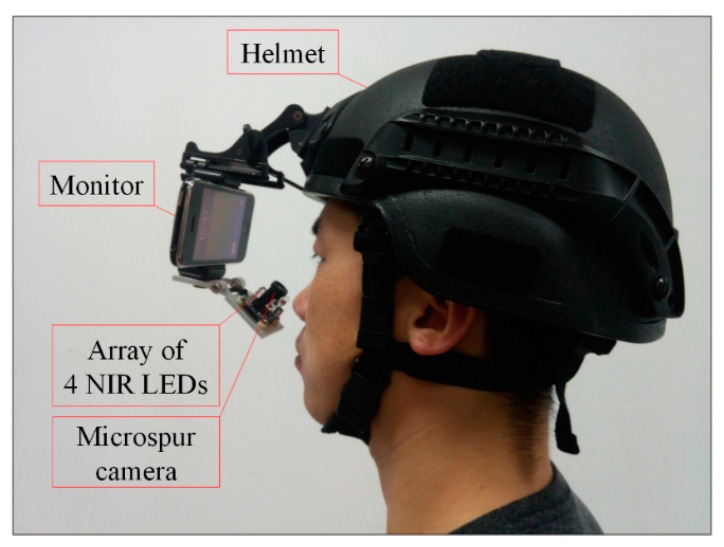
Proposed gaze tracking device.

In this study, we develop a wearable gaze tracking device composed by a helmet, a monitor, an array of four near-infrared light emitting diodes (NIR LEDs) and a microspur camera shown in Figure 1. Considering the imaging distance is limited between 3~5 cm, a microspur camera is adopted to acquire eye image. The image resolution is 640 × 480 pixels (CCD sensor). The wavelength of NIR LED is 850 nm and the power is less than 5mw. The experimental system brings no harm to human eyes [24].

### 2.2. Pupil Detection

#### 2.2.1. Binarization and Opening-and-Closing Operation

**Figure 2:**
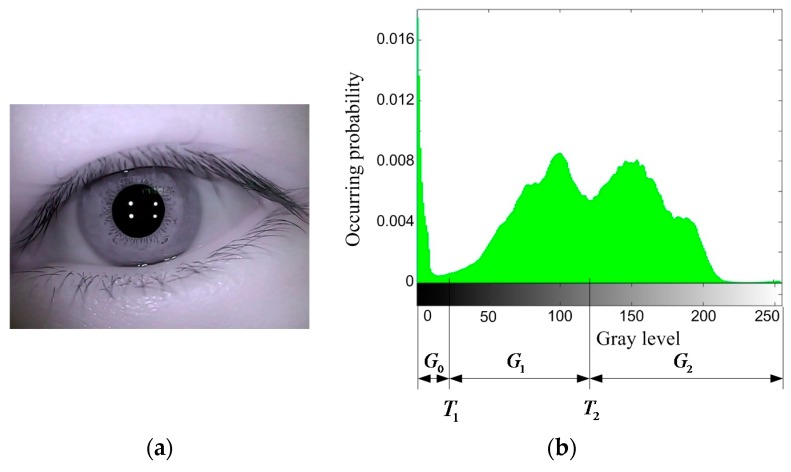
(**a**) Original eye image; (**b**) Gray-level histogram of eye image.

An improved Otsu method is employed to obtain eye binary image in this paper. Proposed by Otsu first in 1979, the Otsu method is based on adaptive threshold selecting [25]. The original eye image is shown in Figure 2a. Gray-level histogram of eye image is shown in Figure 2b.

Assuming number of pixels with gray level i is $n_{i}$ in eye image, all gray levels are divided into 3 groups, as shown in Figure 2b:

(1) $$\begin{array}{l} G_{0}=\left\{0\sim T_{1}\right\}\\ G_{1}=\left\{T_{1}+1\sim T_{2}\right\}\\ G_{2}=\left\{T_{2}+1\sim 255\right\} \end{array}$$

Group $G_{0}$ contains mainly gray levels of black area such as pupil and eyelashes. Group $G_{1}$ contains mainly gray levels of iris and shadows. Group $G_{2}$ contains mainly gray levels of cornea and skin around. Assuming the respective occurring probability of $G_{0}$, $G_{1}$, $G_{2}$ is $\omega_{0}$, $\omega_{1}$, $\omega_{2}$, the corresponding gray level is $\;h_{0}$, $\;\;h_{1}$, $\;\;h_{2}$:

(2) $$\{\begin{array}{l} \omega_{0}=\overset{T_{1}}{\underset{i=0}{\sum }}p_{i},\;h_{0}=\frac{\overset{T_{1}}{\underset{i=0}{\sum }}ip_{i}}{\omega_{0}}\\ \omega_{1}=\overset{T_{2}}{\underset{i=T_{1}+1}{\sum }}p_{i},\;h_{1}=\frac{\overset{T_{2}}{\underset{i=T_{1}+1}{\sum }}ip_{i}}{\omega_{1}}\\ \omega_{2}=\overset{255}{\underset{i=T_{2}+1}{\sum }}p_{i}=1-\omega_{0}-\omega_{1},\;h_{2}=\frac{\overset{255}{\underset{i=T_{2}+1}{\sum }}ip_{i}}{\omega_{2}}=\frac{h-\omega_{0}h_{0}-\omega_{1}h_{1}}{\omega_{2}} \end{array}$$

$p_{i}=n_{i}/N$ is the occurring probability of each gray level. $N=\overset{255}{\underset{i=0}{\sum }}n_{i}g\left(T_{1},T_{2}\right)$ is the total pixel number. $h=\overset{255}{\underset{i=0}{\sum }}ip_{i}$ is average gray level of eye image.

The class variances are defined as

(3) $$\{\begin{array}{l} \sigma_{0}^{2}=\overset{T_{1}}{\underset{i=0}{\sum }}\frac{{\left(i-h_{0}\right)}^{2}p_{i}}{\omega_{0}}\\ \sigma_{1}^{2}=\overset{T_{2}}{\underset{i=T_{1}+1}{\sum }}\frac{{\left(i-h_{1}\right)}^{2}p_{i}}{\omega_{1}}\\ \sigma_{2}^{2}=\overset{255}{\underset{i=T_{2}+1}{\sum }}\frac{{\left(i-h_{2}\right)}^{2}p_{i}}{\omega_{2}} \end{array}$$

The within-class variance is defined as

(4) $$\sigma_{W}^{2}=\omega_{0}\sigma_{0}^{2}+\omega_{1}\sigma_{1}^{2}+\omega_{2}\sigma_{2}^{2}$$

We develop an improved and fast solution method of optimal thresholds. According to Equations (3) and (4), within-class variance is transformed into integral form in Equation (5).

(5) $$\sigma_{W}^{2}=\int_{0}^{T_{1}}\frac{{\left(i-h_{0}\right)}^{2}p_{i}}{\omega_{0}}+\int_{T_{1}+1}^{T_{2}}\frac{{\left(i-h_{1}\right)}^{2}p_{i}}{\omega_{1}}+\int_{T_{2}+1}^{255}\frac{{\left(i-h_{2}\right)}^{2}p_{i}}{\omega_{2}}$$

Partial derivative on $T_{1}$ and $T_{2}$ is calculated respectively on both sides of Equation (5). The calculation result is shown in Equation (6).

(6) $$\{\begin{array}{l} 2T_{1}-h_{0}-h_{1}=0\\ 2T_{2}-h_{1}-h_{2}=0 \end{array}$$

Formula to solve threshold in Otsu method is expanded as Equation (7).

(7) $$\text{g}\left(T_{1},T_{2}\right)=\text{Arg}\;\underset{0<T_{1}<T_{2}<255}{\text{Max}}\left\{\omega_{0}{\left(h_{0}-h\right)}^{2}+\omega_{1}{\left(h_{1}-h\right)}^{2}+\omega_{2}{\left(h_{2}-h\right)}^{2}\right\}$$

According to Equations (6) and (7), optimal thresholds can be solved. For each pixel point in the original eye image, mean gray-level of a 3 × 3 neighboring region around it is calculated to substitute its original gray-level. The occurring probabilities of new gray-levels are calculated and utilized to solve optimal segmentation threshold $T_{1}$ and $T_{2}$ according to Equations (6) and (7). According to the distributing regularity of eye image’s gray-level histogram, value of $T_{1}$ is limited between 0~50, value of $T_{1}$ is limited between $T_{1}$~150. The maximum value of $\text{g}\left(T_{1},T_{2}\right)$ is calculated according to Equation (7) and the corresponding $\left(T_{1},T_{2}\right)$ is the optimal threshold solved.

The computational complexity of the new method to solve optimal threshold is decreased. As shown in Table 1, the segmentation time of improved method is less than that of original Otsu, which contributes to the real-time quality of eye gaze tracking.

**Table 1:** Segmentation time.

Method	Original Otsu	Improved Otsu
Time/ms	32.4	17.1

**Figure 3:**
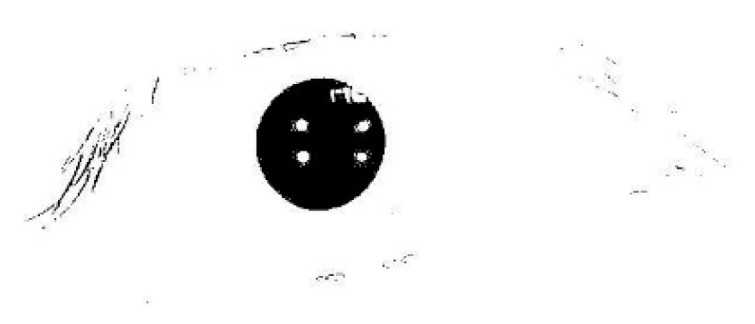
Eye’s binary image with Otsu optimal threshold.

In order to extract the pupil, threshold $T_{1}$ is utilized in the process of binarization. Eye’s binary image is shown in Figure 3.

**Figure 4:**
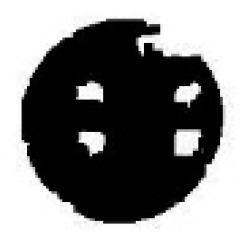
Result of opening-and-closing operation.

To eliminate interference points (mainly remnant eyelashes and eyelids) clearly, opening-and-closing operation with structure elements of different size are employed. According to the shape and size of interference factors shown in Figure 3, a 0.3$T_{1}$ × 0.3$T_{1}$ square structure element is utilized in the process of opening operation, and a 0.7$T_{1}$ × 0.7$T_{1}$ square structure element is utilized in the process of closing operation. The operating result is shown in Figure 4.

#### 2.2.2. Rough Location of Pupil Area and Center

**Figure 5:**
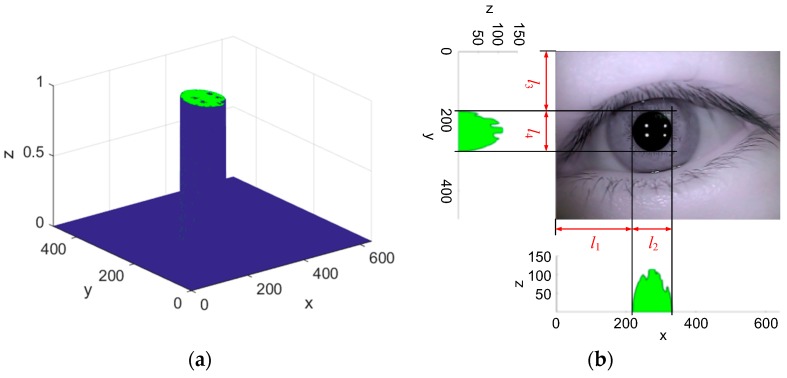
(**a**) 3D gray-level histogram of opening-and-closing operation result; (**b**) Rough location of pupil area and center.

Pupil image acquired through opening-and-closing operation presents an elliptical shape (irregular at glints and natural light reflection). 3D gray-level histogram of opening-and-closing operation result is shown in Figure 5a. Projection along x and y axis of 3D gray-level histogram is shown in Figure 5b. Rough location of pupil area and center position is determined by distribution of gray level in projection image. Rough pupil area locates in a rectangular box with length $l_{2}$ and width $l_{4}$. Estimated pupil center is defined as $o_{p}^{\prime }=(l_{1}+l_{2}/2,l_{3}+l_{4}/2)$. Estimated pupil radius is defined as $r_{p}^{\prime }=(l_{2}+l_{4})/4$.

#### 2.2.3. Collection of Pupil Boundary Points

A novel circular ring rays location (CRRL) method is proposed for pupil boundary points detection based on a modified Starburst. The proposed method has the following advantages than the original Starburst method. First, a series of rays are shot from inner circular ring to outer circular ring in proposed method instead of shooting rays from a guessed point to detected point. In original Starburst, a second shooting of rays is needed to collect more pupil counter candidates. In our proposed method, shooting rays once can collect sufficient pupil boundary points to fit ellipse, which saves the period of pupil boundary points collection. The style of shooting rays can also save calculation time because the rays shot are shorter than those shot in original Starburst method. Second, RANSAC is utilized in original Starburst to distinguish and separate pupil contour points (inliers) and interference points (outliers), which costs much time. We calculate the gradient amplitude at pixels neighboring pupil boundary utilizing pixel gray values of pupil and iris region in advance. Then a threshold of gradient amplitude is set to detect pupil boundary points. Number of pupil boundary points detected on each ray is counted to eliminate interference points. The experimental results show that the method for interference points elimination is suitable and effective in CRRL method. Third, cubic spline interpolation is utilized neighboring collected pupil boundary points to determine subpixel-precise pupil boundary points, which contributes to the accuracy enhancement of pupil center location.

Collection steps of pupil boundary points are presented in detail below:

**Input:** Gray-level eye image.

**Output:** Point set of pupil boundary points.

**Figure 6:**
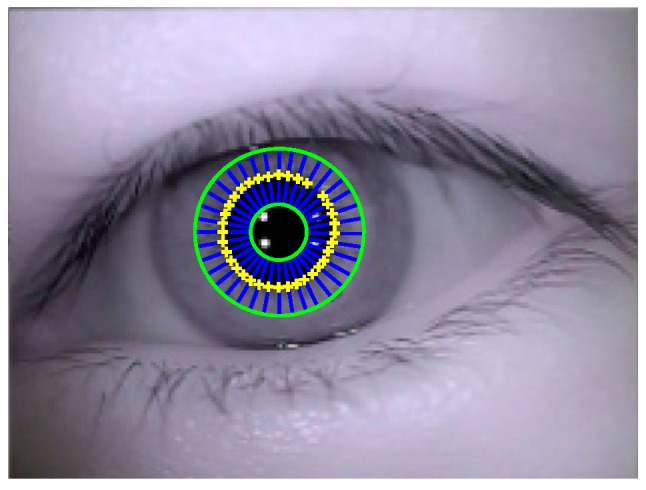
Extraction result of pupil boundary points.

Step 1: Building of circular ring area. As shown in Figure 6, in order to build a circular ring area including pupil boundary inside, estimated pupil center $o_{p}^{\prime }$ is taken as center of inner and outer ring (green line) with respective radius $0.5r_{p}^{\prime }$ and $1.5r_{p}^{\prime }$.

Step 2: Location of pupil boundary points. 36 rays (with equal gap $10^{{}^{\circ}}$) are shot from inner to outer circular ring. Gradient $\nabla f={\left[\;\begin{array}{cc} {\text{g}}_{x} & {\text{g}}_{y} \end{array}\right]}^{\text{T}}$ is calculated at each pixel location (x,y) along shooting direction of each ray. $M\left(x,y\right)=\sqrt{{\text{g}}_{x}^{\;2}+{\text{g}}_{y}^{\;2}}$ is calculated as gradient amplitude. According to variation range of gradient amplitude neighboring pupil contour, a threshold of gradient amplitude is set as $∆\in \left[1.3\delta ,1.5\delta \right]\;\left(\delta =T_{2}-T_{1}\right)$ in advance to select pupil boundary points. If gradient amplitude at pixel location (x,y) along shooting direction is within the range of ∆, pixel (x,y) is recorded as one of pupil boundary points. Located pixel points matching threshold δ on each ray are counted.

Step 3: Elimination of interference points. When interference factors (glints and natural light reflection) are located on or around pupil contour, number of pixels matching threshold δ on the ray may be more than 1. In this case, all boundary points recorded on the ray are eliminated to avoid interference caused by glints and natural light reflection.

Step 4: Subpixel-precise location of pupil boundary points. To enhance location accuracy of pupil boundary points, cubic spline interpolation [26] is utilized neighboring collected pupil boundary points in Step 2 to determine subpixel-precise pupil boundary points.

Step 5: Mark of pupil boundary points. As shown in Figure 6, determined pupil boundary points are marked with yellow “+”. All the determined candidates of pupil boundary points are collected into one point set for ellipse fitting.

#### 2.2.4. Ellipse Fitting

Total least squares (TLS) [27,28] was proposed first in 1980. An improved total least squares is developed in this paper to fit collected pupil boundary points. Compared with least squares (LS) method, errors of independent and dependent variable are taken into account in the calculating process of total least squares. In TLS, matrix equation Ax=b is solved by considering errors in both data matrix A and observation vector b. To compensate errors existed in A and b, perturbation vector e is utilized to perturb observation vector b, and simultaneously, perturbation matrix E is utilized to perturb observation data matrix A. Both e and E are of minimum amount.

Assuming the elliptic equation of eye pupil is $Ax^{2}+Bxy+Cy^{2}+Dx+Ey+F=0$, constraint condition is set as A+C=1 [29] in order to obtain higher fitting accuracy. Then elliptic equation is deformed as Equation (8).

(8) $$Bx_{i}y_{i}+C\left(y_{i}^{2}-x_{i}^{2}\right)+Dx_{i}+Ey_{i}+F=-x_{i}^{2}$$

where i=1,2,⋯,n, n is the number of pupil boundary points extracted. Errors in pixel position (x,y) is defined as $\left(v_{x},v_{y}\right)$, the ideal form of Equation (8) is defined as

(9) $$B\left(x_{i}y_{i}-v_{x_{i}y_{i}}\right)+C\left[\left(y_{i}^{2}-x_{i}^{2}\right)-\left(v_{y_{i}}^{2}-v_{x_{i}}^{2}\right)\right]+D\left(x_{i}-v_{x_{i}}\right)+E\left(y_{i}-v_{y_{i}}\right)+F=-\left(x_{i}^{2}-v_{x_{i}}^{2}\right)$$

Transform Equation (8) into matrix form

(10) Mτ=Y

where $M=[\begin{array}{ccccc} x_{1}y_{1} & y_{1}^{2}-x_{1}^{2} & x_{1} & y_{1} & 1\\ x_{2}y_{2} & y_{2}^{2}-x_{2}^{2} & x_{2} & y_{2} & 1\\ \vdots & \vdots & \vdots & \vdots & \vdots \\ x_{i}y_{i} & y_{i}^{2}-x_{i}^{2} & x_{i} & y_{i} & 1\\ \vdots & \vdots & \vdots & \vdots & \vdots \\ x_{n-1}y_{n-1} & y_{n-1}^{2}-x_{n-1}^{2} & x_{n-1} & y_{n-1} & 1\\ x_{n}y_{n} & y_{n}^{2}-x_{n}^{2} & x_{n} & y_{n} & 1 \end{array}]$, $\tau ={\left[\begin{array}{ccccc} B & C & D & E & F \end{array}\right]}^{\text{T}}$, $\;Y={\left[\begin{array}{ccccccc} -x_{1}^{2} & -x_{2}^{2} & \cdots & -x_{i}^{2} & \cdots & -x_{n-1}^{2} & -x_{n}^{2} \end{array}\right]}^{\text{T}}$. Let augmented matrix H=[−Y,M] and its singular values $\sigma_{1}\geq \sigma_{2}\geq \cdots \geq \sigma_{\text{min}}$) are calculated utilizing SVD method. According to the subspace interpretation of total least squares, the total least squares solution of matrix equation Mτ=Y is deduced as

(11) $$\tau_{\text{TLS}}={\left(M^{\text{T}}M-\sigma_{\text{min}}^{2}\text{I}\right)}^{-1}M^{\text{T}}Y$$

where $\sigma_{\text{min}}$ is the minimal singular value of augmented matrix H. Consequently, $\sigma_{\text{min}}^{2}$ is the common variance of each component in perturbation matrix D=[−e,E].

For the reason that row of constant in coefficient matrix M cannot be considered in SVD, we propose an improved method for SVD solution. By setting $\alpha_{1i}=x_{i}y_{i}$, $\alpha_{2i}=y_{i}^{2}-x_{i}^{2}$, $\alpha_{3i}=x_{i}$, $\alpha_{4i}=y_{i}$, $\beta_{i}=-x_{i}^{2}$, error equation of ellipse can be define as

(12) $$v_{i}=Bx_{1i}+Cx_{2i}+Dx_{3i}+Ex_{4i}+F-z_{i}$$

Here we set

(13) $$\;\{\begin{array}{l} {\bar{\alpha }}_{r}=\frac{1}{n}\overset{n}{\underset{i=1}{\sum }}X_{ri}\;\left(r=1,2,3,4\right)\\ \bar{\beta }=\frac{1}{n}\overset{n}{\underset{i=1}{\sum }}\beta_{i} \end{array}$$

Therefore, coefficient F is described as

(14) $$F=\bar{\beta }-{\bar{\alpha }}_{1}B-\alpha_{2}C-{\bar{\alpha }}_{3}D-{\bar{\alpha }}_{4}E=\bar{\beta }-{\bar{\alpha }}^{T}\tau^{\prime }$$

where $\bar{\alpha }={\left[\begin{array}{cccc} {\bar{\alpha }}_{1} & {\bar{\alpha }}_{2} & {\bar{\alpha }}_{3} & {\bar{\alpha }}_{4} \end{array}\right]}^{T}$, $\tau^{\prime }={\left[\begin{array}{cccc} B & C & D & E \end{array}\right]}^{T}$.

By taking Equation (14) into Equation (12), we acquire

(15) $$\varepsilon =X\tau^{\prime }-Z$$

where $\varepsilon =\left[\begin{array}{c} v_{1}\\ v_{2}\\ \vdots \\ v_{n} \end{array}\right]$, $X=\left[\begin{array}{cccc} x_{11}-{\bar{x}}_{1} & x_{21}-{\bar{x}}_{2} & \cdots & x_{41}-{\bar{x}}_{4}\\ x_{12}-{\bar{x}}_{1} & x_{22}-{\bar{x}}_{2} & \cdots & x_{42}-{\bar{x}}_{4}\\ \vdots & \vdots & \cdots & \vdots \\ x_{1n}-{\bar{x}}_{1} & x_{2n}-{\bar{x}}_{2} & \cdots & x_{4n}-{\bar{x}}_{4} \end{array}\right]$, $\tau^{\prime }=\left[\begin{array}{c} B\\ C\\ D\\ E \end{array}\right]$, $Z=\left[\begin{array}{c} z_{1}-\bar{z}\\ z_{2}-\bar{z}\\ \vdots \\ z_{n}-\bar{z} \end{array}\right]$.

The total least squares solution of matrix equation $\varepsilon =X\tau^{\prime }-Z$ is described as

(16) $$\tau_{\text{TLS}}^{\prime }={\left(X^{\text{T}}X-\gamma_{\text{min}}^{2}I\right)}^{-1}X^{\text{T}}Z$$

New augmented matrix is defined as L=[−Z,X]. In order to improve the fitting accuracy and stability of TLS, a novel and fast SVD solution method is utilized to acquire the singular values of matrix L. L is described as SVD format in Equation (17).

(17) $$L=U\Sigma V^{\text{T}}$$

Matrix Q is defined as

(18) $$Q=L^{\text{T}}L={\left(U\Sigma V^{\text{T}}\right)}^{\text{T}}\left(U\Sigma V^{\text{T}}\right)=\left(V\Sigma U^{\text{T}}\right)\left(U\Sigma V^{\text{T}}\right)=V\Sigma^{2}V^{\text{T}}$$

Equation (19) shows the multiplication result of different rows $L_{s},L_{t}$ in matrix L.

(19) $$Q_{st}={\left[L_{s},L_{t}\right]}^{\text{T}}\left[L_{s},L_{t}\right]$$

where 1≤s≤4, 1≤t≤4, s≠t. Eigenvalue matrix $\Sigma_{st}$ is calculated as

(20) $$\Sigma_{st}=\Delta_{st}^{\text{T}}(Q_{st})\Delta_{st}$$

Then rows of matrix L are redefined as $\left[L_{s},L_{t}\right]\Delta_{st}$. Orthogonal transformation is conducted for any two redefined rows of matrix L. Non-diagonal elements of matrix Q are eliminated. Eigenvalue matrix of Q is solved as

(21) $$\Sigma^{\prime }=V^{\text{T}}QV=V^{\text{T}}\left(L^{\text{T}}L\right)V=\left[\begin{array}{cccc} \gamma_{1}^{2} & & & 0\\ & \gamma_{2}^{2} & & \\ & & \ddots & \\ 0 & & & \gamma_{\text{m}}^{2} \end{array}\right]$$

$\gamma_{1},\gamma_{2},\cdots ,\gamma_{\text{m}}$ ($\gamma_{1}\geq \gamma_{2}\geq \cdots \geq \gamma_{\text{m}}$) are the singular values of matrix L. $\tau^{\prime }={\left[\begin{array}{cccc} B & C & D & E \end{array}\right]}^{\text{T}}$ is calculated according to Equation (16). Pupil center can be acquired through Equation (22).

(22) $$\;\{\begin{array}{c} x_{p}=\frac{BE-2CD}{4AC-B^{2}}\\ y_{p}=\frac{BD-2AE}{4AC-B^{2}} \end{array}$$

where A=1−C.

The sensitivity of the TLS problem depends on the ratio $r=\left({\tilde{\sigma }}_{p}-\sigma_{p+1}\right)/{\tilde{\sigma }}_{p}^{\prime }$. ${\tilde{\sigma }}_{p}$, $\sigma_{p+1}$ and ${\tilde{\sigma }}_{p}^{\prime }$ are the respective least singular value of matrix X(or M), L(or H) and $X_{0}$(or $M_{0}$)(coefficient matrix in corresponding LS problem). When the value of ratio r is larger, the TLS will be more accurate than LS. During the ellipse fitting of pupil boundary points, the respective ratios r of TLS problem solved by SVD and improved SVD are 0.82 and 0.94. The improved TLS achieves a higher accuracy than original TLS. Improved TLS method makes a compensation for errors in pixel location. The fitting result is more closed to the ideal form of elliptic equation (Equation (9)).

**Figure 7:**
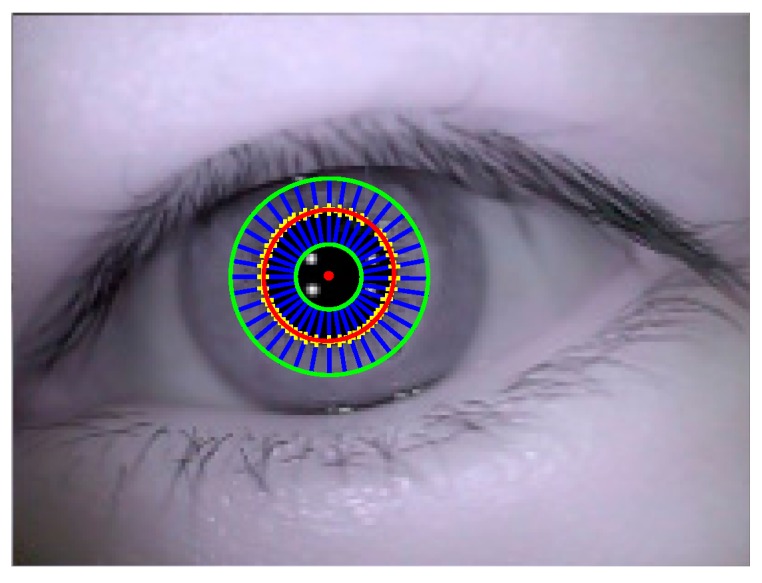
Ellipse fitting result.

The result of ellipse fitting is shown in Figure 7. Red ellipse represents the fitted pupil contour. Red “•” represents the center of fitted pupil contour.

### 2.3. Glint Detection

For the reason that the pixel number of glint region is limited and there is halo existing around glint contour, the proposed method for pupil detection is not suitable for glint. Improved Gaussian fitting is utilized to locate glint center.

#### 2.3.1. Rough Location of Glint Region

**Figure 8:**
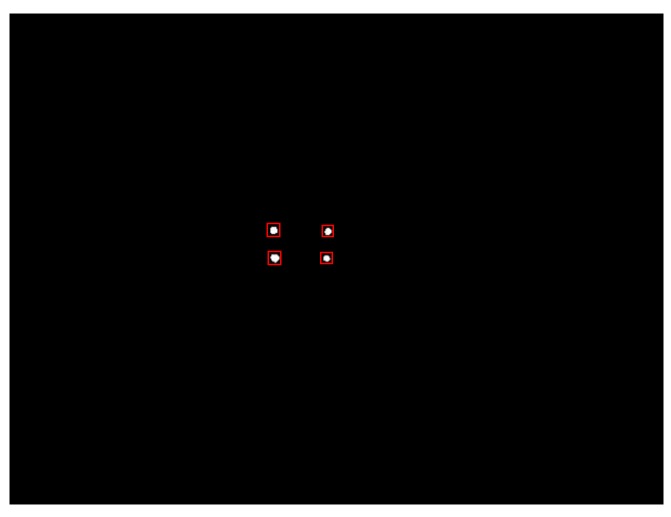
Rough location of glint region.

Because illumination intensity of glint is higher and its gray-levels are near to 255, threshold=240 is adopted on binarization of eye image to extract glints. A 2 × 2 square structure element is utilized in the process of opening-and-closing operation to filter binary image. As shown in Figure 8, red rectangular boxes are utilized to locate rough glint regions.

#### 2.3.2. Gaussian Fitting

**Figure 9:**
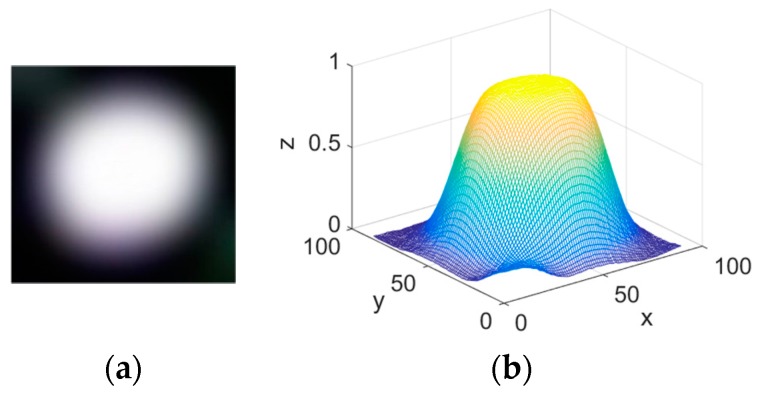
(**a**) Enlarged glint region; (**b**) 3D gray-level histogram of enlarged glint.

Figure 9a shows the enlarged glint region. The 3D gray-level histogram of enlarged glint is shown in Figure 9b. The glint’s illumination intensity suits for Gaussian distribution [30].

Gaussian function of glint illumination intensity is defined as Equation (23):

(23) $$I\left(x,y\right)=H\cdot e^{-\left[\frac{{\left(x-x_{\text{g}}\right)}^{2}}{2\sigma_{x}^{2}}+\frac{{\left(y-y_{\text{g}}\right)}^{2}}{2\sigma_{y}^{2}}\right]}$$

I(x,y) is the gray-level of pixel (x,y) in glint region. As the amplitude of Gaussian distribution, H is the highest gray-level in glint region. $\left(x_{\text{g}}\;,y_{\text{g}}\right)$ represents the glint center to be calculated. $\;\sigma_{x}$ an $\;\sigma_{y}$ is the respective standard deviation of gray-level in horizontal and vertical direction. A logarithmic operation is conducted to Equation (23). The arrangement and deformation result is as follow:

(24) $$z=ax^{2}+by^{2}+cx+dy+e$$

where z=lnI(x,y), $\;a=-1/2\sigma_{x}^{2}$, $b=-1/2\sigma_{y}^{2}$, $c=x_{\text{g}}/\sigma_{x}^{2}$, $\;d=y_{\text{g}}/\sigma_{y}^{2}$, $\;e=-x_{\text{g}}^{2}/2\sigma_{x}^{2}-y_{\text{g}}^{2}/2\sigma_{y}^{2}+\text{ln}H$. Subpixel-precise boundary points of glint are extracted with cubic spline interpolation neighboring glint contour. Pixel points inside glint contour are substituted into Equation (24) for calculating. The improved total least squares proposed in Section 2.2.4 is utilized for the solution of overdetermined equations composed by Equation (24). Glint center is calculated according to Equation (25) with the solved value of a,  b,  c,  d.

(25) $$\{\begin{array}{c} x_{\text{g}}=-\frac{c}{2a}\\ y_{\text{g}}=-\frac{d}{2b} \end{array}$$

**Figure 10:**
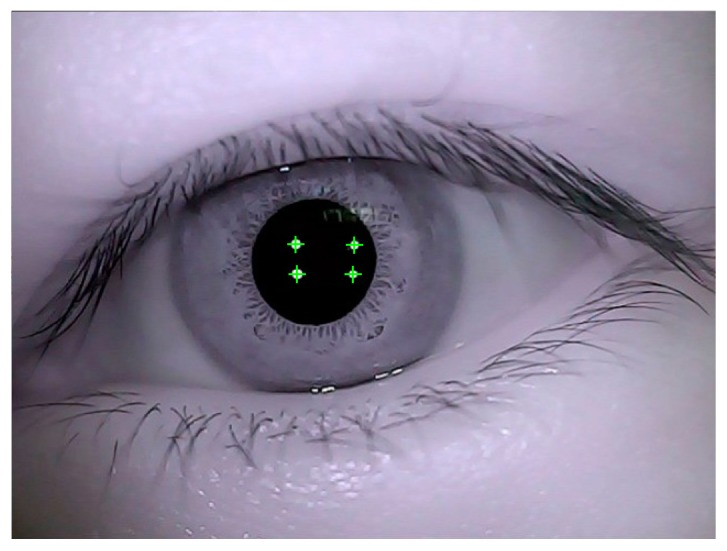
Detection result of glint center.

Figure 10 shows the detection result of glint center (marked with green “+”).

## 3. Experimental Results

### 3.1. Pupil Detection

#### 3.1.1. Pupil Detection of Single Subject

**Figure 11:**
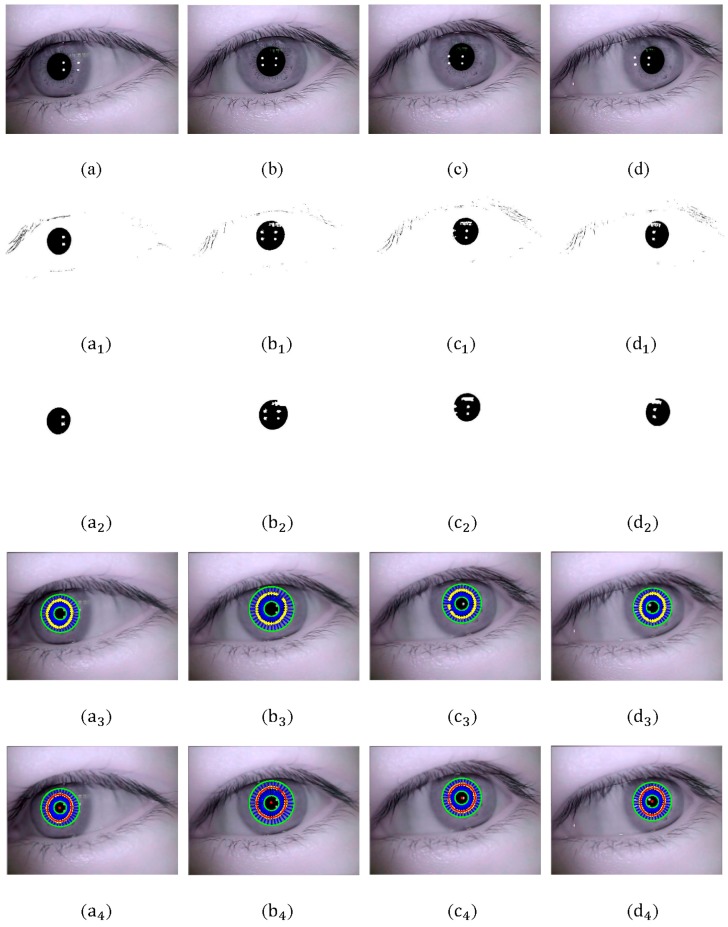
(**a**–**d**) Original eye image; (**a_1_**–**d_1_**) Eye binary image utilizing improved Otsu optimal threshold; (**a_2_**–**d_2_**) Results of opening-and-closing operation; (**a_3_**–**d_3_**) Extraction result of pupil boundary points; (**a_4_**–**d_4_**) Pupil fitting result.

The process of pupil detection is shown in Figure 11. Figure 11a-d shows four original eye image with different relative position of pupil and glints acquired from single subject; ${\text{a}}_{1}$-${\text{d}}_{1}$ shows eye binary image utilizing improved Otsu optimal threshold; ${\text{a}}_{2}$-${\text{d}}_{2}$ shows result of opening-and-closing operation with 5 × 5 square structure element; ${\text{a}}_{3}$-${\text{d}}_{3}$ shows extraction result of pupil boundary points (marked with yellow “+”); ${\text{a}}_{4}$-${\text{d}}_{4}$ shows fitting results of pupil (red ellipse). The center of fitted pupil contour is marked with red “∂”.

Table 2 shows the parameters of pupil detection, including threshold $T_{1}$ and $T_{2}$, rough pupil center and final pupil center fitted.

**Table 2:** Parameters of pupil detection.

Eye Image	Threshold $T_{1}$	Threshold $T_{2}$	Rough Pupil Center ($x_{0},y_{0}$)	Final Pupil Center ($x_{p},y_{p}$)
Figure 11a	13	117	(199, 230)	(196.39, 230.88)
Figure 11b	15	120	(311, 212)	(310.66, 209.67)
Figure 11c	15	122	(344, 194)	(344.34, 192.55)
Figure 11d	14	119	(379, 207)	(378.43, 206.37)

#### 3.1.2. Pupil Detection of Different Subjects

**Figure 12:**
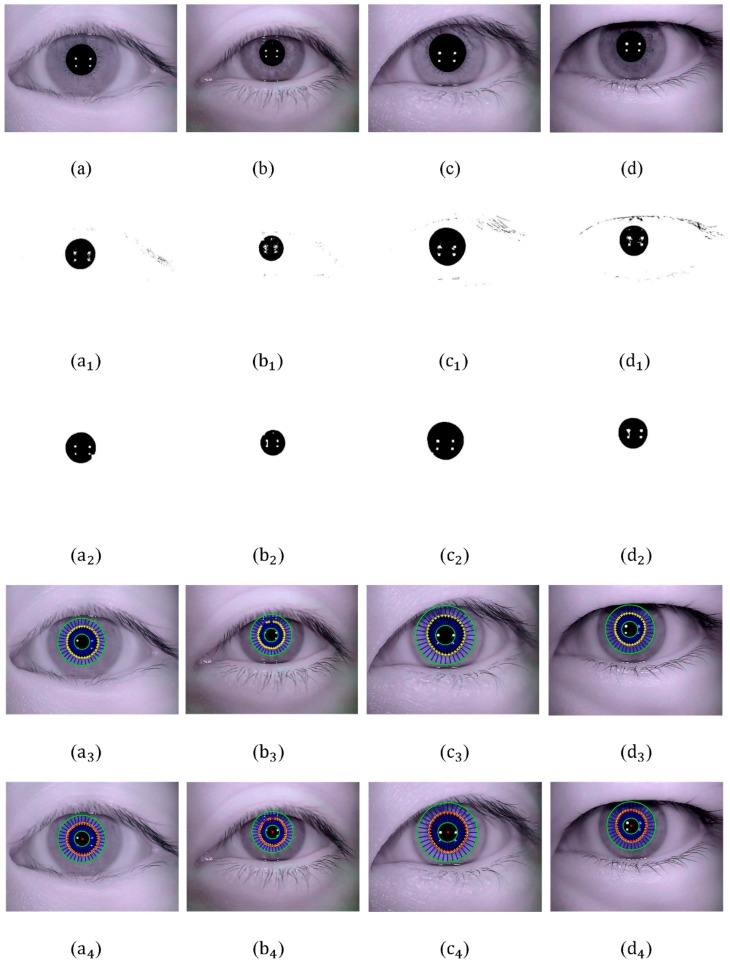
(**a**–**d**) Original eye image; (**a_1_**–**d_1_**) Eye binary image utilizing improved Otsu optimal threshold; (**a_2_**–**d_2_**) Results of opening-and-closing operation; (**a_3_**–**d_3_**) Extraction result of pupil boundary points; (**a_4_**–**d_4_**) Pupil fitting result.

In order to verify the applicability of the proposed circular ring rays location(CRRL) method, original eye images of another four different subjects are acquired. The experimental results are shown in Figure 12. In Section 2.2.1, a larger size of structure element is set in process of closing operation than that in opening operation. For subjects with heavy eyelashes and eyelids, different sizes of structure element in opening-and-closing operation can ensure the complete elimination of remnant interference factors caused by eyelashes and eyelids.

Table 3 shows the parameters of pupil detection, including threshold $T_{1}$ and $T_{2}$, rough pupil center and final pupil center fitted.

**Table 3:** Parameters of pupil detection.

Eye Image	Threshold $T_{1}$	Threshold $T_{2}$	Rough Pupil Center ($x_{0},\;\;y_{0}$)	Final Pupil Center ($x_{p},\;\;y_{p}$)
Figure 12a	22	132	(282, 210)	(284.12, 211.65)
Figure 12b	13	116	(318, 186)	(317.46, 185.34)
Figure 12c	24	133	(292, 186)	(293.59, 185.60)
Figure 12d	11	121	(299, 164)	(299.38, 162.11)

### 3.2. Glint Detection

**Figure 13:**
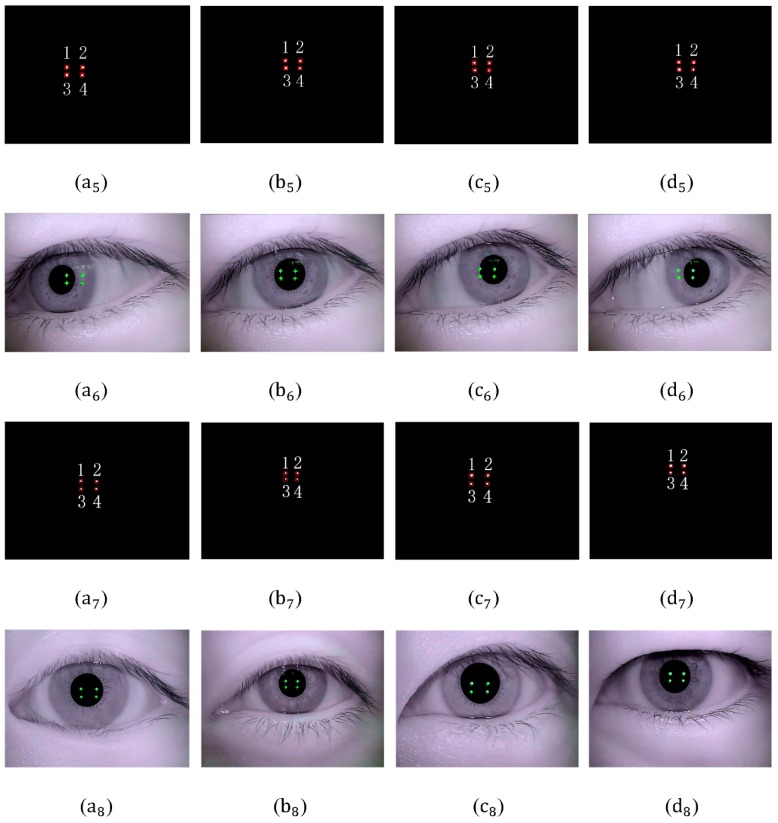
(**a_5_**–**d_5_**) Rough location of glints in Figure 11a–d; (**a_6_**–**d_6_**) Glint detection results in Figure 11a–d; (**a_7_**–**d_7_**) Rough location of glints in Figure 12a–d; (**a_8_**–**d_8_**) Glint detection results in Figure 12a–d.

Glint detection is implemented for Figure 11a–d and Figure 12a–d. The process of detection is shown in Figure 13. Figure 13${\text{a}}_{5}$–${\text{d}}_{5}$,${\text{a}}_{7}$–${\text{d}}_{7}$ show the rough location of glints after binarization and filtering operation. No. 1,2,3,4 are glint number defined. Figure 13${\text{a}}_{6}$–${\text{d}}_{6}$,${\text{a}}_{8}$–${\text{d}}_{8}$ show the detection result of glints. The glint center is marked with green “+”.

Table 4 shows the parameters of glint detection in Figure 11a–d and Figure 12a–d.

**Table 4:** Parameters of glint detection.

	Detected Glint Center ($x_{\text{g}},\;\;y_{\text{g}}$)			
Glint Number	1	2	3	4
Figure 11a	(212.39, 214.42)	(268.28, 214.64)	(213.53, 241.31)	(266.76, 241.24)
Figure 11b	(293.85, 201.79)	(345.34, 202.49)	(294.15, 227.63)	(343.94, 227.71)
Figure 11c	(296.90, 191.21)	(348.58, 191.36)	(298.34, 217.17)	(347.55, 217.43)
Figure 11d	(314.53, 196.17)	(366.49, 197.12)	(316.03, 221.18)	(365.87, 222.52)
Figure 12a	(264.25, 207.31)	(318.64, 208.15)	(265.12, 235.43)	(317.20, 235.98)
Figure 12b	(211.39, 133.26)	(252.13, 134.68)	(221.64, 149.52)	(251.54, 149.22)
Figure 12c	(265.47, 186.29)	(321.40, 186.24)	(263.68, 216.44)	(319.87, 215.31)
Figure 12d	(284.31, 152.37)	(331.82, 152.21)	(283.14, 176.33)	(329.13, 176.45)

### 3.3. Stability and Error

To evaluate the stability and accuracy of proposed method, 105 eye images of each subject are acquired for pupil and glint detection. Stability, RMS error and processing time of proposed method in this paper are shown in Table 5. As a reference, stability, RMS error and processing time of detection methods in paper [13,20,21,22] are listed in Table 5. As can be seen from the experimental results in Table 5, stability, accuracy and real-time quality of the proposed method are better than those in paper [13,20,21,22].

**Table 5:** Stability, RMS error and processing time of different methods.

Method	Pupil Detection			Glint Detection		
	Stability	Error (Pixels)	Time (ms)	Stability	Error (Pixels)	Time (ms)
Proposed method	99.4%	2.17	43.6	98.7%	0.69	21.5
Paper [13]	94.9%	6.48	92.1	90.9%	1.73	38.6
Paper [20]	95.2%	7.86	65.5	94.1%	1.28	34.1
Paper [21]	97.9%	5.43	54.3	-	-	-
Paper [22]	96.6%	5.95	126.4	-	-	-

## 4. Conclusions

A novel and robust method of pupil and glint detection using a wearable camera sensor and near-infrared LED array for gaze tracking system is proposed in this paper. A circular ring rays location (CRRL) method is proposed for detection of pupil boundary points. An improved Otsu method is proposed for threshold segmentation. The experimental results show that the segmentation time of improved method is less than that of original Otsu, which contributes to the real-time quality of eye gaze tracking. Size and number of gradient amplitude are employed to eliminate interference factors. In order to compensate for errors of pupil boundary points in horizontal and vertical direction, improved total least squares is developed to fit ellipse. The experimental results show that the improved total least squares has a higher accuracy than original total least squares on pupil ellipse fitting. For the purpose of a higher location accuracy of glint, improved total least squares is utilized for the solution of Gaussian function deformation to calculate glint center. As we can see from the experimental results, stability, accuracy and real-time quality of the proposed method are better than those existing currently for pupil and glint detection. When interference factors such as glints and natural light reflection are located neighboring pupil boundary, interference points caused can be eliminated fast and effectively. The proposed method contributes to the enhancement of stability, accuracy and real-time quality of gaze tracking system.
